# Different Shades of Desmoid-Type Fibromatosis (DTF): Detection of Noval Mutations in the Clinicopathologic Analysis of 32 Cases

**DOI:** 10.3390/diagnostics14192161

**Published:** 2024-09-28

**Authors:** Rana Ajabnoor

**Affiliations:** Department of Pathology, Faculty of Medicine, King Abdulaziz University and King Abdulaziz University Hospital, Jeddah 22252, Saudi Arabia; rana.ajabnoor@gmail.com or rajabnoor@kau.edu.sa

**Keywords:** desmoid-type fibromatosis, histologic pattern, beta-catenin immunostain, non-CTNNB1 gene mutations

## Abstract

Background: Desmoid-type fibromatosis (DTF) is a locally aggressive myofibroblastic/fibroblastic neoplasm with a high risk of local recurrence. It has a variety of histologic features that might confuse diagnosis, especially when detected during core needle biopsy. The Wnt/β-catenin pathway is strongly linked to the pathogenesis of DT fibromatosis. Method: This study examined 33 desmoid-type fibromatoses (DTFs) from 32 patients, analyzing its clinical characteristics, histologic patterns, occurrence rates, relationship with clinical outcomes, immunohistochemical and molecular findings. Results: The DTFs exhibit a range of 1 to 7 histologic patterns per tumor, including conventional, hypercellular, myxoid, hyalinized/hypocellular, staghorn/hemangiopericytomatous blood vessels pattern, nodular fasciitis-like, and keloid-like morphology. No substantial association was found between the existence of different histologic patterns and the clinical outcome. All thirty-three (100%) samples of DTF had a variable percentage of cells that were nuclear positive for β-catenin. An NGS analysis detected novel non-CTNNB1 mutations in two DTFs, including BCL10, MPL, and RBM10 gene mutations. Conclusions: This study reveals a diverse morphology of DTFs that could result in misdiagnosis. Therefore, surgical pathologists must comprehend this thoroughly. Also, the importance of the newly identified non-CTNNB1 gene mutations is still unclear. More research and analyses are needed to completely grasp the clinical implications of these mutations.

## 1. Introduction

Desmoid-type fibromatosis (DTF), also referred to as aggressive fibromatosis or desmoid tumor, is a neoplasm characterized by deeply seated, locally aggressive, and infiltrating myofibroblastic/fibroblastic growth. It is considered to have intermediate malignant potential and exhibits a high susceptibility to local recurrence. Notably, DTF does not possess the ability to metastasize, meaning it does not spread to distant sites in the body [1]. DTF is uncommon, representing less than 3% of soft tissue tumors, with an annual incidence of 3–5 cases per million people [2,3,4,5,6,7]. It is more prevalent in women and has a wide age range, with a peak age incidence between the 30s and 40s [5,8,9]. It is anatomically classified as extra-abdominal, abdominal wall (abdominal), and intra-abdominal (deep pelvic and mesenteric) [10,11,12,13,14,15].

The pathogenesis of desmoid fibromatosis involves multiple signaling pathways, including Wnt/β-catenin; Hedgehog (Hh); Notch; Janus-activated kinase (JAK) and signal transducer and activator of transcription (STAT); PI3 Kinase/AKT and mTOR; the transforming growth factor-ß (TGF-ß) regulatory pathway; and estrogen-driven signaling pathways [16]. Among these different pathways, the Wnt/β-catenin pathway is strongly linked to the pathogenesis of DT fibromatosis [17]. The canonical Wnt/β-catenin pathway is responsible for the regulation of cell proliferation, differentiation, apoptosis, and homeostasis and is involved in stem cell maintenance [16,18,19]. CTNNB1 is a proto-oncogene, located on chromosome 3p22.1, that functions as a coactivator within the Wnt/β-catenin signaling pathway, leading to the production of β-catenin protein [20,21]. It is well known that the canonical Wnt/β-catenin pathway is extremely conservative and rigorously regulated [22]. Upon activation, this system promotes the stability of β-catenin and its subsequent translocation to the nucleus. Ultimately, this process facilitates the expression of genes that play a role in cell proliferation, survival, and differentiation. When the Wnt/β-catenin pathway is inactivated, the cytoplasmic β-catenin protein is subjected to degradation by a multiprotein destruction complex. This complex is composed of axin, adenomatous polyposis coli protein (APC), casein kinase 1α (CK1α), and glycogen synthase kinase 3β (GSK3β) [22,23].

It has been established that abnormal activating signaling in the Wnt/β-catenin pathway is implicated in 95% of cases of desmoid fibromatosis [24]. This abnormal activation can be attributed to either a germline mutation that leads to the loss of function of the tumor suppressor gene APC in the context of familial adenomatous polyposis (FAP), or somatic mutations occurring in the β-catenin gene (CTNNB1) [25]. In all scenarios, β-catenin will undergo nuclear translocation and thereafter exhibit an aberrant activation of target genes [24]. Despite affecting the same pathway, both types of mutations are mutually exclusive; therefore, they possess diagnostic utility [2]. The immunohistochemical (IHC) assay can be utilized to identify the nuclear accumulation of β-catenin, which acts as a diagnostic tool for distinguishing desmoid-type fibromatosis (DTF) from other fibrous tumors in soft tissues [26].

The occurrence of desmoid-type fibromatosis is predominantly sporadic, accounting for approximately 90% of cases, and it is linked to somatic mutation in the CTNNB1 gene. On the other hand, 5–10% of desmoid-type fibromatosis cases are hereditary and observed in individuals with familial adenomatous polyposis (FAP), which is characterized by a germline mutation in the adenomatous polyposis coli gene (APC) [27,28,29]. Individuals diagnosed with FAP face a significantly elevated risk, approximately 1000 times greater, of having desmoid-type fibromatosis. Furthermore, within the population of people affected by FAP, an estimated 5–16% will ultimately acquire desmoid-type fibromatosis [27,29]. A very uncommon subset of desmoid fibromatosis, ranging from 5.1% to 9.3%, lacks CTNNB1 or APC gene mutations and is referred to as “wild type” [30,31]. A significant majority (over 85%) of sporadic desmoid-type fibromatosis cases exhibit missense mutations in exon 3 of the CTNNB1 proto-oncogene. These mutations primarily affect the serine threonine phosphorylation sites located at codons 41 and 45. The most observed deleterious alterations include the p. Thr41Ala (T41A) mutation, occurring in 54.4% to 59% of cases, the p. Ser45Phe (S45F) mutation, occurring in 19.6% to 22% of cases, and the p. Ser45Pro (S45P) mutation, occurring in 8.8% to 12% of cases [24,31,32]. At the histopathologic level, DTF consists of a proliferation of fibroblasts/myofibroblasts that exhibit a bland appearance. These cells are grouped in elongated sweeping fascicles, interspersed with delicate thin-walled blood vessels, all set against a collagenous background. Nevertheless, it is not uncommon for DTF to exhibit a range of histologic patterns, including myxoid, hyalinized/hypocellular, hypercellular, nodular fasciitis-like, and keloid-like morphology [15]. These patterns can significantly complicate the diagnosis, particularly when detected during core needle biopsy. Hence, this study involved the analysis of 32 cases of desmoid-type fibromatosis (DTF) with respect to their clinical characteristics, histologic patterns, frequency of occurrence, and their correlation with clinical outcomes as well as immunohistochemical findings. Additionally, this study aims to describe the identification of a novel pathogenic non-CTNNB1 mutation in two cases that were subjected to molecular analysis using next-generation sequencing.

## 2. Materials and Methods

### 2.1. Patients and Samples

**Figure 1:**
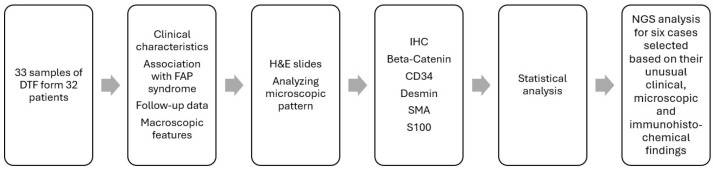
Schematic illustration of the study method. DTF, desmoid-type fibromatosis; FAP, familia adenomatous polyposis; H&E, hematoxylin and eosin; IHC, immunohistochemistry; NGS, next-generation sequencing.

**Figure 2:**
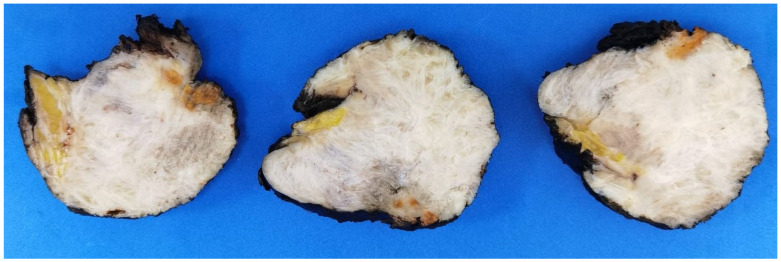
Macroscopically, DTF usually presents as a large infiltrative firm lesion with a trabeculated cut surface.

An ethical approval to conduct this study was obtained from the unit of biomedical ethics at King Abdulaziz University Hospital, Jeddah, Saudi Arabia, reference number 287-23, 23 May 2023. A total of 33 formalin-fixed paraffin-embedded (FFPE) tissue samples of desmoid-type fibromatosis (DTF) were retrieved from the histopathological archives of King Abdulaziz University Hospital spanning the period from 2010 to 2022. These samples were collected from 32 patients. The medical records of patients were reviewed to assess clinical characteristics, age, location, sex, follow-up data, and association with familial adenomatous polyposis (FAP) syndrome, along with the macroscopic features. All samples’ H&E and immunohistochemical slides were thoroughly analyzed and evaluated based on the specific criteria outlined below. The results were statistically analyzed using STATA software version 18, StataCorp LLC. 2023. Stata Statistical Software: Release 18, College Station, TX, USA. Six cases were molecularly tested using next-generation sequencing (NGS), and they were selected to be sequenced based on their unusual clinical and microscopic/immunohistochemical findings (Figure 1). The Macroscopic features of DTF usually show an infiltrative firm gritty lesion with a white, tan trabeculated cut surface, (Figure 2).

### 2.2. Microscopic Features

**Figure 3:**
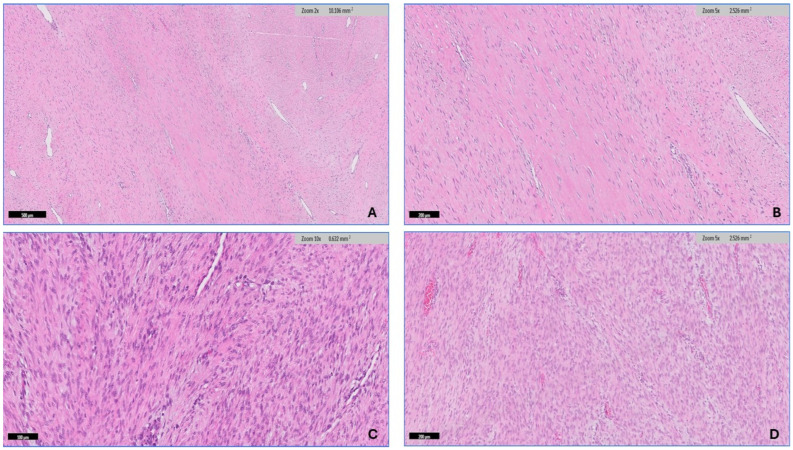
The histologic pattern of desmoid-type fibromatosis. (**A**,**B**) The conventional pattern is characterized by the presence of long, sweeping fascicles of thin fibroblasts/myofibroblasts that are uniformly spaced and exhibit minimal or no cell-to-cell contact. (**C**,**D**) The hypercellular pattern has a higher cellular density compared with the conventional pattern, accompanied by an increase in nuclear overlaps.

**Figure 4:**
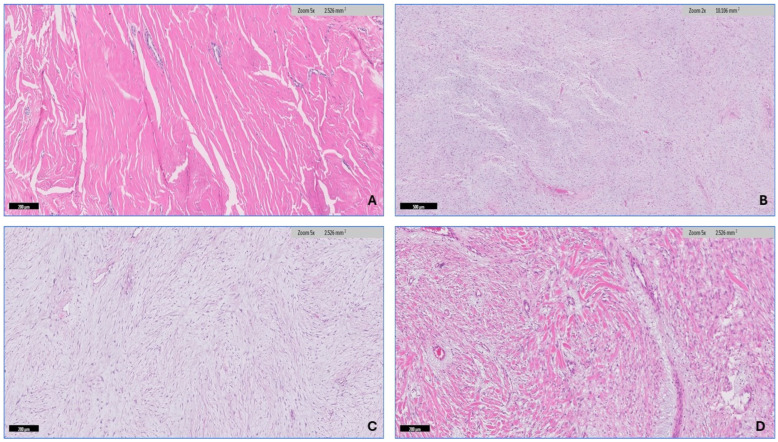
Continued histologic pattern of desmoid-type fibromatosis. (**A**) The hyalinized/hypocellular pattern is characterized by fibroblasts, and myofibroblasts are sparsely distributed within a heavily hyalinized collagenous background. (**B**,**C**) The myxoid pattern is distinguished by a profuse myxoid background including loosely arranged hypocellular regions of spindle cells, resembling cellular myxoma. (**D**) The keloid-like pattern characterized by the presence of collagenized bands that vary in size and exhibit a bright eosinophilic appearance.

**Figure 5:**
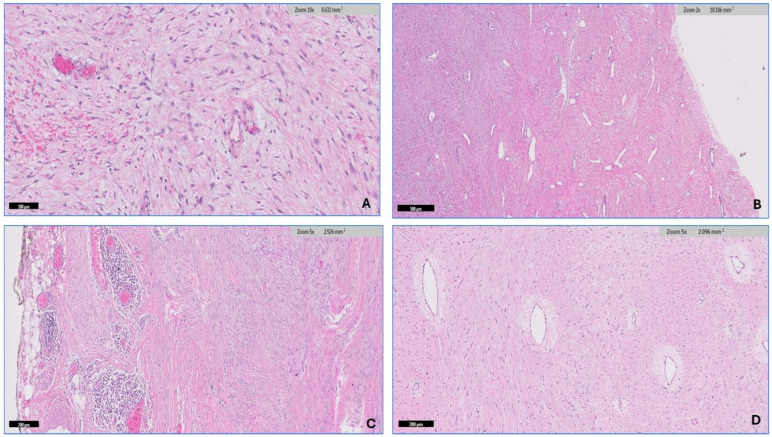
Continued histologic pattern of desmoid-type fibromatosis. (**A**) Nodular fasciitis-like pattern characterized by loose short fascicles of spindle to stellate cells with an edematous background and extravasated RBCs. (**B**) Hemangiopericytomatous-like patten characterized by the presence of thin-walled, branched blood vessels with staghorn-shaped vessels. Additional histological features: (**C**) the presence of lymphoid aggregation, mainly at the periphery of the tumor. (**D**) Perivascular edema.

**Figure 6:**
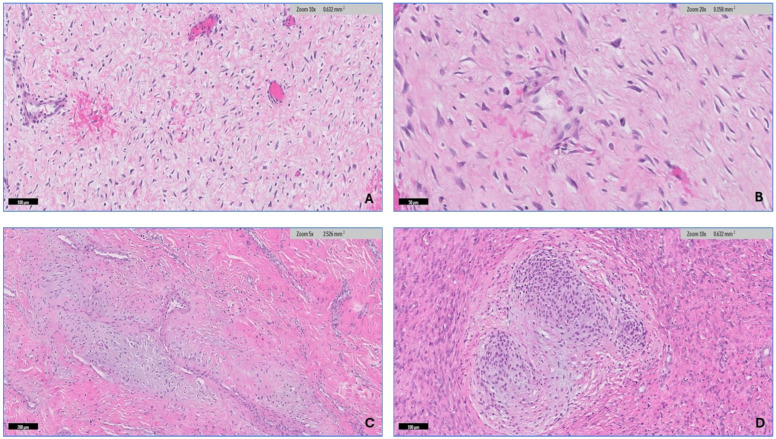
Additional histological features of DTF. (**A**,**B**) Spindle cells with mild to moderate atypia characterized by nuclear hyperchromasia. (**C**,**D**) Two cases showed focal chondromyxoid-like metaplasia.

The soft tissue pathologist (R.M.A.) assessed all H&E-stained slides available for each case, which ranged from 1 to 20 slides. The morphologic diagnosis was then verified through a histological review. After conducting a thorough search of the histopathology literature [15,33], it has been observed that desmoid fibromatosis exhibits seven distinct histologic patterns. These include conventional, hypercellular, hyalinized/hypocellular, myxoid, keloid-like, nodular fasciitis-like, and hemangiopericytomatous-like patterns. Desmoid-type fibromatosis is characterized by the presence of fibroblast/myofibroblast cells that exhibit oval, pale-staining, or vesicular nuclei with one or two small, inconspicuous nucleoli. These cells do not display nuclear hyperchromasia or atypia, possess varying quantities of cytoplasm, and have ill-defined cell membranes. The microscopic descriptions that have been utilized to analyze each pattern in this study are as follows: (1) The conventional pattern is distinguished by uniform cellularity and the presence of long, sweeping fascicles that extend across the diameter of a low power field. These fascicles consist of thin fibroblasts/myofibroblasts that are evenly spaced and exhibit minimal or no cell-to-cell contact (Figure 3A,B). (2) The hypercellular pattern has a higher cellular density compared with the conventional pattern, accompanied by an increase in nuclear overlaps (Figure 3C,D). (3) The hyalinized/hypocellular pattern is characterized by a low number of cells and a reduced cellular density. Fibroblasts and myofibroblasts are sparsely distributed within a heavily hyalinized collagenous background (Figure 4A). (4) The myxoid pattern is distinguished by a profuse myxoid background, including loosely arranged hypocellular regions of spindle cells (Figure 4B,C). (5) The keloid-like pattern is characterized by the presence of collagenized bands that vary in size and exhibit a bright eosinophilic appearance (Figure 4D). (6) A nodular fasciitis-like pattern characterized by loose short and storiform fascicles of spindle to stellate cells with an edematous background and extravasated RBCs (Figure 5A). (7) The hemangiopericytomatous-like pattern, characterized by the presence of thin-walled, branched blood vessels, with a particular emphasis on the presence of staghorn-shaped vessels (Figure 5B). The occurrence and relative proportion of these patterns were documented for each case of desmoid-type fibromatosis in this set of specimens. Additional histologic parameters were examined, which encompassed the presence of lymphoid aggregation (Figure 5C), perivascular edema (Figure 5D), tumor boundaries, spindle cell atypia characterized by nuclear hyperchromasia, and an increased N/C ratio (Figure 6A,B), along with the quantification of mitotic figures. A univariate analysis was conducted to examine the correlation between the histologic pattern and recurrence of DTF.

### 2.3. Immunohistochemistry

The 33 FFPE tissue blocks, which were fixed in 10% neutral-buffered formalin, were sliced into sections that were 4 μm thick. Each section was then stained with β-catenin, CD34, desmin, smooth muscle actin (SMA), and S100 ready-to-use and IVD use mouse monoclonal antibodies. The specific antibody sources, clones, and concentrations can be found in Table 1. The immunohistochemistry procedure was conducted utilizing the Bench-Mark XT automated equipment manufactured by Ventana Medical Systems, Inc., located in Tucson, AZ, USA. This is a fully automated processing of barcode-labeled slides that included baking of the slides; solvent-free deparaffinization; antigen retrieval with a cell conditioning solution CC1 (mild: 36 min conditioning and standard: 60 min conditioning) and pre-primary peroxidase; incubation with the mouse monoclonal antibodies for 32 min at 37 °C; and application of ultraView™ Universal DAB Inhibitor, ultraView Universal DAB Chromogen, ultraView Universal DAB H_2_O_2_, and ultraView Universal DAB Copper. Counterstaining with hematoxylin (2021) and post-counter staining with bluing reagent (2037) were performed for a maximum of 4 min. After staining, the sections were dehydrated in ethanol, cleared in xylene, and covered with DPX mounting medium and cover slips. In every immunostaining procedure, both positive and negative controls were incorporated. The expressions of β-catenin, CD34, desmin, SMA, and S100 were assessed using a quantitative method by analyzing the number of stained tumor cells and the localization of a specific reaction.

### 2.4. Next-Generation Sequencing (NGS) DNA and RNA Sequencing

An NGS analysis was conducted using a commercially available solid tumor panel by Centogene, Am Strande 7, 18055 Rostock, Germany. DNA extraction from the FFPE material was performed manually using a commercially available kit that combines deparaffinization, enzymatic steps, column base purification followed by DNA quantification, and quality control. Genomic DNA was enzymatically fragmented, and regions of interest were enriched using DNA capture probes. The final indexed libraries were sequenced on an Illumina NGS platform (Nextseq 550). For the solid tumor panel (sequencing; somatic analysis), the coding regions of the panel genes, 2 bp of flanking intronic sequences, and known pathogenic/likely pathogenic variants within these genes (coding and non-coding) were targeted for analysis. The panel gene list that was analyzed included the following genes: ABL1, AKT1, AKT2, AKT3, APC, AR, ARID1A, ASXL1, ATM, ATR, ATRX, BAP1, BRAF, BRCA1, BRCA2, CDH1, CDK12, CDK4, CDKN1B, CDKN2A, CDKN2B, CHEK1, CHEK2, CREBBP, CSF1R, CTNNB1, DDR2, EGFR, ERBB2, ERBB3, ERBB4, EZH2, FANCA, FANCD2, FANCI, FBXW7, FGFR1, FGFR2, FGFR3, FGFR4, GNA11, GNAQ, GNAS, HNF1A, HRAS, IDH1, IDH2, KDR, KEAP1, KIT, KMT2A, KMT2C, KMT2D, KRAS, MAP2K1, MAP2K2, MEN1, MET, MLH1, MPL, MRE11, MSH2, MSH6, MTOR, NBN, NF1, NF2, NFE2L2, NOTCH1, NOTCH2, NOTCH3, NRAS, NTRK3, PALB2, PDGFRA, PIK3CA, PIK3R1, PMS2, POLE, PTCH1, PTEN, PTPN11, RAD50, RAD51, RAD51B, RAD51C, RAD51D, RB1, RBM10, RET, RIT1, RNF43, SETD2, SLX4, SMAD4, SMARCA4, SMARCB1, SMO, SPOP, SRC, STK11, TP53, TSC1, TSC2, TSHR, and VHL; for hotspot analysis: ALK, ARAF, AXL, BTK, CBL, CCND1, CDK6, ERCC2, ESR1, FLT3, FOXL2, GATA2, H3-3A, HIST1H3B, JAK1, JAK2, JAK3, KNSTRN, MAGOH, MAP2K4, MAPK1, MAX, MDM4, MED12, MYC, MYCN, MYD88, NTRK1, NTRK2, PDGFRB, PIK3CB, PPP2R1A, RAC1, RAF1, RHEB, RHOA, ROS1, SF3B1, STAT3, TERT, TOP1, U2AF1, and XPO1. Data analysis, including alignment to the hg19 human reference genome (Genome Reference Consortium GRCh37), variant calling, and annotation was performed using validated software.

## 3. Results

### 3.1. Clinical Features

In this study, the cohort comprised 33 samples of desmoid-type fibromatosis (DTF) from 32 patients, consisting of 22 females (69%) and 10 males (31%). The median age at the time of diagnosis was 35.5 years, with an average (mean) age of 38.42 years (ranging from 3 to 92 years). Desmoid-type fibromatosis was observed in a range of anatomical locations. To facilitate the analysis within this cohort, it was categorized into three subtypes: abdominal wall (6 cases, 18.18%), intra-abdominal (8 cases, 24.24%), and extra-abdominal (19 cases, 57.58%). Furthermore, the identified extra-abdominal sites of DTF are the legs, thighs, shoulders, neck, mandibular and sub-mental regions, parotid glands, back, breast and axilla (Table 2). Tumor size information was available for 25 samples, with an average size of 8.83 cm, ranging from 1 to 26 cm. Twenty-six DTFs were excised and seven were biopsied, with no available information about the type of surgical excision. Out of the total number of patients, clinical follow-up data were obtained for eighteen individuals, representing 56% of the sample. The median duration of follow-up was 15.5 months, with a range of 1 to 131 months. Among these patients, seven experienced recurrences of the DTF (39%), and one individual succumbed as a result of the disease. The cause of death was attributed to a large intra-abdominal desmoid-type fibromatosis (DTF), which led to severe colonic obstruction.

### 3.2. Histopathologic Findings

The cohort of DTF tumors exhibit a range of 1 to 7 histologic patterns per tumor, with a mean of 3.57 and a median of 3. The conventional pattern was the most common, occurring in all 33 tumors (100%), with a percentage of total tumor volume ranging from 5 to 100% (mean 59%, median 70%). The hypercellular pattern is the next most common pattern in this collection, appearing in 23 tumors (67%) and ranging from 5 to 50% (mean 18%, median 10%) of the total tumor volume. The myxoid pattern is observed in 20 tumors (60%), with volumes ranging from 5 to 40% (mean 15.25%, median 10%). The hyalinized/hypocellular patten ranged from 5 to 80% (mean 21.05%, median 15%) of the total tumor volume in 19 (57.5%) tumors. Sixteen (48.5%) tumors have staghorn/hemangiopericytomatous blood vessels that range from 5 to 20% (mean 9.1%, median 5%) of the total tumor volume. Twelve (36.3%) tumors have a nodular fasciitis-like pattern with a volume range of 5 to 60% (mean 13.75%, median 5%) of the overall tumor volume. The keloid-like pattern is found in seven (21.2%) of the tumors and accounts for 5 to 20% (mean 9.3%, median 5%) of the overall tumor volume. No substantial association was found between the existence of different histologic patterns and the clinical outcome (Table 3).

Perivascular edema was found in 31 (94%) of the tumors, while lymphoid follicles were found in 18 (54.5%) tumors. Cytological atypia was found in 27 (87%) samples, ranging from mild to moderate in 22 (67%) and 5 (15.15%) tumors, respectively. Mitosis was found in thirteen (39.3%) samples, ranging from one to four mitosises/ten high-power fields (HPFs), with a mean of two and a median of one. Two cases showed rare findings of chondromyxoid metaplasia (Figure 6C,D).

Among all tumors, 61% displayed irregular borders, 24.24% had clearly defined borders, and 15.15% could not be assessed due to insufficient information about the excisional specimen. Among the eighteen cases of DTF that were followed up on, positive margins were observed in eleven samples (61%) and negative margins were observed in four samples (22.2%), and information about the excisional specimen was not available for three cases.

### 3.3. Immunohistochemistry

**Figure 7:**
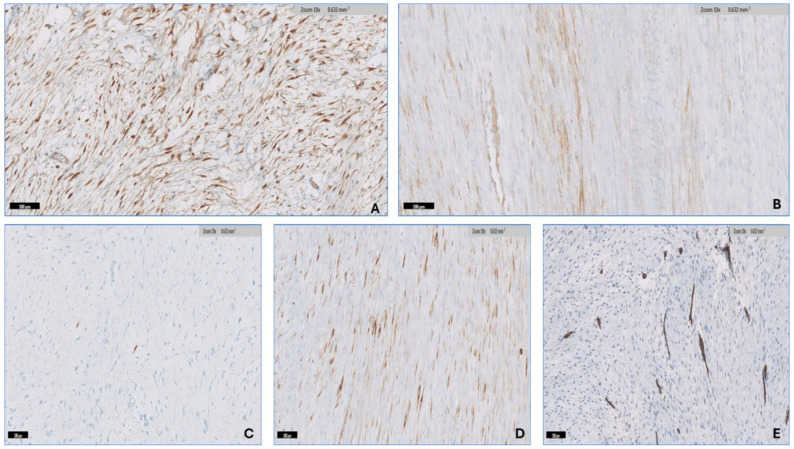
Immunohistochemistry of DTF. (**A**) β-catenin shows diffuse nuclear positivity in spindle cells. (**B**) SMA shows focal positivity in the spindle cells. (**C**) S100 shows rare positivity in spindle cells. (**D**) Desmin shows focal positivity in the spindle cells. (**E**) CD34 is negative in the spindle cells.

The percentage of cells with nuclear positivity for β-catenin ranged from 10 to 90% (mean 66.4%, median 80%) in all thirty-three (100%) DTF samples (Figure 7A). SMA positivity was found in 28 (85%) of the tumors, with percentages of positive cells ranging from 10 to 70% (mean 36.6%, median 32.5%) (Figure 7B). S100 was found in 17/33 tumors (51.5%), with the percentage of positive cells ranging from 1 to 5% (mean 2.4%, median 1%) (Figure 7C). Desmin was detected in 12/33 samples (36.36%), with the percentage of positive cells ranging from 10 to 30% (mean 14.16%, median 10%) (Figure 7D). CD34 was found in 3/33 tumors (9.1%), with the percent of positivity ranging from 10 to 30% (mean 17%, median 10%) (Figure 7E).

### 3.4. Molecular Findings

**Figure 8:**
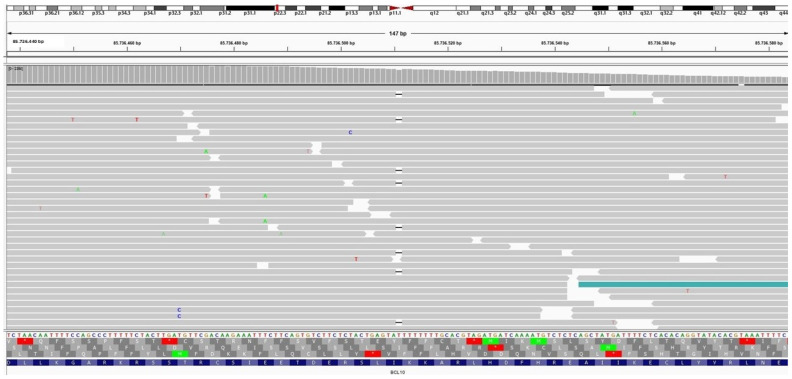
A non-CTNNB1 mutation of BCL10 mutation variant c.136del p.(Ile46Tyrfs 24) (Frequency: 5.9% of 1649 NGS reads) causes a disruption in the reading frame, beginning at codon 46.

**Figure 9:**
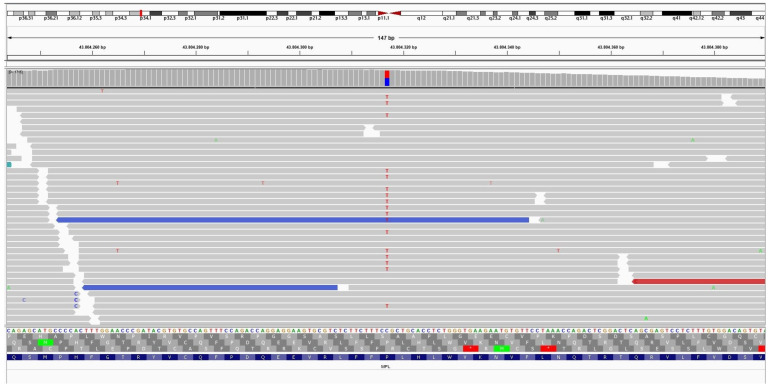
A non-CTNNB1 mutation of MPL gene mutation variant c.317C>T p.(Pro106Leu) (Frequency: 48.4% of 1544 NGS reads).

The NGS analysis identified a deleterious mutation in the CTNNB1 gene variation c.114_149del p.(Ala39_Gly50del) (Frequency 16.4% of 1846 NGS reads) in a 24-year-old female patient with abdominal wall desmoid-type fibromatosis. This mutation is an in-frame deletion of 36 base pairs in exon 3, resulting in the loss of 12 amino acid residues in the N-terminal region of the β-catenin gene. In addition, it was discovered that in the same case, a non-CTNNB1 mutation, including BCL10 mutation variant c.136del p.(Ile46Tyrfs 24) (Frequency: 5.9% of 1649 NGS reads) (Figure 8), causes a disruption in the reading frame, beginning at codon 46 along with MPL gene mutation variant c.317C>T p.(Pro106Leu) (Frequency: 48.4% of 1544 NGS reads) (Figure 9). A 46-year-old male patient with Crohn’s disease and a small 1 cm intrabdominal desmoid-type fibromatosis (DTF) was found to have a CTNNB1 mutation variant c.133T>C p.(Ser45Pro) (Frequency: 21.1% of the 3287 NGS reads) in the N-terminus of the protein. Additionally, the patient had an RBM10 gene mutation variant c.1049_1067inv p.(Gln350_Ser356delinsArgAlaLeu) (Frequency: 5.4% of the 1065 NGS reads). An example of extra-abdominal (right leg) DTF in a 37-year-old male patient with an abundance of myxoid, hyalinized, and hypocellular histologic pattern and limited conventional pattern NGS identified the CTNNB1 variant c.121A>G p.(Thr41Ala) (Frequency: 40% of 1889 NGS reads) situated in the protein’s N-terminus. The APC gene mutation variant c.4348C>T p.(Arg1450) (Frequency: 66% of the 1338 NGS reads) generates a premature stop codon in one of the two FAP cases of DTF that have been studied, one of which is a 10-year-old girl with a neck mass and recurrent abdominal wall mass. In another case of a 37-year-old female patient with FAP syndrome and intra-abdominal DTF, NGS revealed three APC genetic alterations, including: 1. c.3316G>T p.(Gly1106) (Frequency: 51% of 2059 NGS reads); 2. c.4604del p.(Asn1535Metfs 30) (Frequency: 14% of 1665 NGS reads); and 3. c.4645dup p.(Gln1549Profs 10) (Frequency: 8% of 1700 NGS reads). One case of breast DTF NGS failed to reveal CTNNB1 or APC gene mutation (CTNNB1 wild type).

## 4. Discussion

Desmoid-type fibromatosis (DTF) is a benign fibroblastic tumor that tends to be locally aggressive. It is most frequently found in adults aged 20–44 years, with a higher prevalence in females. The number of female cases is 2.2 to 3.9 times greater than the number of male cases [3,7,13,34,35]. Our findings in this study align well with this assertion, as we observed a higher prevalence of DTF among adults with an average age of 38.42 years, primarily among females. The increased incidence of DTF in women may be attributed to the underlying influence of estrogen, as seen by the accelerated tumor growth observed during pregnancy and the use of oral contraceptive pills (OCPs), while regression occurs during menopause [16,34,35].

Trautmann et al. examined 204 cases of desmoid-type fibromatosis (DTF) and found that it was most prevalent in extra-abdominal regions (*n* = 106/204, 58%), particularly in sporadic instances; intra-abdominal sites ranked second (*n* = 59/204, 32%); and abdominal wall sites were the least prevalent (*n* = 19/204, 10%) [31]. In line with the findings of Jiyeon An et al. in their examination of 70 DTF cases, there was a higher prevalence of extra-abdominal sites (*n* = 52, 74.3%) [36]. These findings align with the results of this study, which identified extra-abdominal sites as the most prevalent locations (*n* = 19/33, 57.58%), followed by intra-abdominal sites (eight cases, 24.24%) and the abdominal wall (six cases, 18.18%). Previous research has documented a range of DTF sizes from 0.7 to 29 cm, with a mean of 7 cm. The current study’s findings align with this range, as the DTF sizes vary from 1 to 26 cm with an average of 8.83 cm [15,31].

In a study analogous to the current one, Zreik et al. classified seven unique histologic patterns of desmoid fibromatosis and observed no significant association between any of them and clinical outcomes [15]. Out of the seven patterns observed, the conventional pattern was the most common, being present in all 165 DTFs (100%) with a percentage of total tumor volume varying from 10 to 100 percent, which corresponds with the results of this study. In contrast to the findings of the present study, which reported the hypercellular pattern as the second most common, occurring in 23 out of 33 cases (67%) and comprising a range of 5% to 50% of the total tumor volume, Zreik et al. observed the hyalinized/hypocellular pattern as the second most prevalent (46 cases, 28%), comprising a range of 5% to 80% of the total tumor volume [15]. In contrast, the hypercellular pattern was the least frequently observed in their study, comprising six cases (4%) and spanning a range of 5% to 80% of the total tumor volume.

In the diagnostic process of DTF, the hypercellular pattern may lead to erroneous diagnosis. For instance, if the hypercellular pattern is exclusively sampled from the DTF in a core needle biopsy, it will prompt the following as a potential differential diagnosis: solitary fibrous tumor (SFT), monophasic synovial sarcoma, low-grade dedifferentiated liposarcoma, peripheral nerve sheath tumor, NTRK fusion-associated mesenchymal neoplasms including NTRK rearranged sarcoma, infantile fibrosarcoma and lipofibromatosis-like neural tumor (LPF-NT), myofibroma, adult type fibrosarcoma, low-grade leiomyosarcoma, and spindle cell rhabdomyosarcoma.

From a histological perspective, SFTs are diverse cellular tumors consisting of ovoid to spindled cells that grow in a patternless or storiform pattern against a collagenous to myxoid background. These tumors frequently contain perivascular fibrosis and thin-walled, large branching blood vessels that resemble “staghorn”-shaped (HPC) blood vessels [37]. With the aid of immunohistochemistry, it is possible to differentiate between SFT and DTF as CD34 has been reported as positive in 90–95% of SFTs [38,39], unlike in DTF, in which CD34 has always been reported as negative [40,41]. In the current cohort, CD34 was only expressed in 3/33 (9%) of DTFs with a range of 10 to 30% of positivity in the tumor cells. A STAT6 IHC stain has emerged as a useful surrogate marker of NAB2–STAT6 gene fusion of the molecular signature of SFTs with excellent sensitivity and specificity [39,42,43,44,45]. However, weak nuclear positivity of STAT6 was reported by Demico et al. in 68% of DTFs, and robust nuclear positivity was observed in 8% of DTFs [46]. Positive nuclear β-catenin staining was also found in 22–40% of solitary fibrous tumors [47,48]. Therefore, in difficult instances, molecular testing via NGS to detect β-catenin mutations or the targeted hotspot fusion of NAB2–STAT6 is utilized to confirm the diagnosis.

Synovial sarcoma (SS) is categorized into three subtypes: monophasic (majority of the cases), biphasic (BSS), and poorly differentiated. If only a hypercellular area of desmoid fibromatosis is sampled in a core needle biopsy, the monophasic synovial sarcoma (MSS) should be considered as a possible diagnosis in the differential. The MSS typically consists of uniform and monotonous spindle cells that are organized in fascicles or compact sheets [46]. The spindle cells exhibit a high nucleus-to-cytoplasm ratio, with nuclei that are hyperchromatic and overlapping and nucleoli that are inconspicuous [49] The tumor stroma exhibited varying quantities of hyalinized collagen [49]. The BSS comprises a combination of spindle and epithelial tumor cells. SS is characterized by distinctive SS18::SSX fusions (SS18::SSX1, SS18::SSX2, and SS18::SSX4) that arise from a chromosomal translocation at locus X;18 (p11;q11). The application of cutting-edge next-generation immunohistochemistry to the SS18-SSX chimeric protein (E9X9V) and the C-terminus of SSX (E5A2C) for the diagnosis of synovial sarcoma demonstrated excellent sensitivity and specificity [50]. Alternatively, molecular testing or fluorescence in situ hybridization (FISH) may be utilized to detect this genetic alteration, thereby contributing to the diagnosis of SS [50].

Dedifferentiated liposarcoma (DDL) mainly arises in the retroperitoneum and infrequently in the deep soft tissue of the extremities [51]. From a histological perspective, DDL typically exhibits an abrupt distinction between well-differentiated regions of mature fat, such as well-differentiated liposarcoma (WDL)/atypical lipomatous tumor (ALT), and dedifferentiated regions [51]. The dedifferentiated components have a diverse range of morphological characteristics that resemble undifferentiated spindle cell sarcoma with variable grades. Low-grade dedifferentiation is characterized by mildly atypical fibroblast-like spindle cells and low mitosis, which may suggest desmoid fibromatosis as a potential alternative diagnosis if this is the sole component observed in a biopsy specimen [51]. Fortunately, the utilization of FISH testing, which achieves a specificity of 95% and sensitivity of 100% for MDM2 gene amplification, facilitates the differentiation of DDL from other spindle cell neoplasms [52].

Peripheral nerve sheath tumors encompass a diverse collection of spindle cell tumors, such as neurofibroma, schwannoma, perineurioma, and malignant peripheral nerve sheath tumors (MPNSTs). Neurofibroma and schwannoma are distinguished by their consistent expression of S100 and SOX10, while perineurioma lacks S100 expression and instead exhibits membranous staining with EMA, GLUT-1, and Claudin-1 [53]. Most MPNSTs are aggressive spindle cell tumors characterized by tapered nuclei and distributed in alternating zones of high and low cellularity, resulting in a marbled appearance. Brisk mitotic activity and necrosis are common, as are heterologous elements on occasion [53]. A subset of MPNSTs are of low grade. MPNSTs exhibit inconsistent expressions of S100 and/or SOX10 and display a lack of H3K27me3 expression [53]. In this study, focal S100 positivity was found in 17/33 of desmoid tumors (51.5%). However, this expression is non-specific and should not affect the diagnosis of DTF, as S100 positivity can also be observed in fibrous scars [48,54].

NTRK fusion-associated mesenchymal neoplasm refers to a diverse group of tumors, such as NTRK rearrangement sarcoma, lipofibromatosis-like neural tumor (LPF-NT), and infantile fibrosarcoma, among others. NTRK-rearranged sarcoma exhibits a wide range of morphological characteristics, spanning from low-grade monomorphic spindle cells organized in intersecting fascicles or a patternless pattern with a low mitotic count to high-grade monomorphic spindle cells displaying a herringbone pattern and frequent mitosis, resembling infantile fibrosarcoma [55,56]. Patterns resembling MPNST, SFT, hemangiopericytomatous, inflammatory myofibroblastic tumor, myxoid, and dermatofibrosarcoma protuberance have been documented in these tumors [55,56]. The LPF-NT is often superficially located and consists of fascicles of mildly to moderately atypical spindle cells with low mitotic count, and it infiltrates the subcutaneous adipose tissue with a honeycomb pattern. NTRK fusion-associated mesenchymal neoplasm exhibits simultaneous expression of S100 and CD34, as well as the presence of NTRK gene fusion identified using FISH or NGS testing [55,56,57].

Myofibroma is a superficial benign myofibroblastic tumor that tends to commonly occur in children. It is a classic myofibroma characterized histologically by a distinct biphasic pattern where the center of the tumor contains immature-looking, plump, spindle cells surrounded by a branching vasculature similar to that of a hemangiopericytoma, whereas the outer part of the tumor consists of nodules and bundles of cells that can appear as either hyalinized, myoid, or chondroid-like cells. The rara variant of myofibroma exhibits high cellularity and atypical features [1]. Classic myofibroma typically expresses SMA in both components and is negative for desmin, whereas cellular myofibroma frequently expresses SMA and desmin [1]. Both forms of myofibromas lack nuclear β-catenin staining [58]. Classic myofibromas usually carry PDGFRB mutations, whereas cellular myofibromas have SRF-RELA gene fusions [1,59,60].

Adult-type fibrosarcoma is an exceptionally uncommon kind of tumor, accounting for less than 1% of all soft tissue tumors in adults [1]. Microscopically, it is consist of monomorphic spindle cells with moderate atypia arrange in a herringbone-like pattern with variable collagen background [48]. The diagnosis is mostly based on the absence of specific immunohistochemical and molecular genetic findings, making it a diagnosis that is reached by exclusion [1].

Low-grade leiomyosarcoma (LMS) is most usually found in deep soft tissue and is composed of spindle-shaped cells with plump, blunt-ended nuclei and a moderate to ample pale to highly eosinophilic fibrillary cytoplasm. The cells are arranged in long crossing fascicles parallel and perpendicular to the plane of section [1]. At least one myogenic marker, such as SMA, desmin, or h-caldesmon, should be positive in LMS using immunohistochemistry [1]. An et al. identified SMA expression in 87.41% of DTFs [36], which is comparable with the findings of the present study in which 28/33 (85%) of DTFs expressed SMA, with positive cell percentages varying from 10% to 70% in a tram-like pattern. Desmin is typically negative in DTFs [61], but in this cohort, it was found in 12/33 DTFs (36.36%), with the percentage of positive cells ranging from 10 to 30%. Leiomyosarcomas lack nuclear β-catenin immunostaining [61], rendering β-catenin a useful diagnostic tool for excluding leiomyosarcoma in challenging cases.

Spindle cell/Sclerosing rhabdomyosarcoma predominantly affects the head and neck, followed by the extremities [1]. Histologically distinguished by relatively monomorphic spindle cell proliferation grouped in long, crossing fascicles on a background of fibrotic, sclerosing, and hyalinized stroma. Occasionally, the tumors may have a fibrosarcoma-like pattern. Nuclear atypia ranges from mild to moderate, with frequent mitotic figures and the presence of occasional rhabdomyoblasts [1]. The tumors were frequently infiltrative in the surrounding skeletal muscle and adipose tissue. The immunohistochemical profile is distinguished by desmin reactivity, which is often diffuse and robust; at least localized reactivity for myogenin, diffuse positivity for MYOD1; and SMA negativity [1]. Ng et al. investigated the expression of β-catenin immunostaining in 17 rhabdomyosarcomas without mentioning subclassification and discovered that 4/17 (23.5%) revealed nuclear expressions ranging from low to high [26]. Focal myogenin positivity was observed in 20% of DTFs, but no MyoD1 reactivity was found [62]. As a result, if DTFs exhibit hypercellular or hypocellular/hyalinized areas, the diagnosis of spindle cell/sclerosing RMS should be considered; however, the interpretation of myogenin and myoD1 should be conducted with caution, paying attention to the pattern and the pitfall, as entrapped degenerating skeletal muscles may test positive for myogenin [62].

Gastrointestinal stromal tumor (GIST) is a significant potential alternative diagnosis for intra-abdominal desmoid-type fibromatosis (DTF). GISTs exhibit a diverse array of histologic characteristics, including spindle cells, epithelioid cells, or a combination of both. Spindle cell GIST is composed of diffuse sheets or an indistinct storiform pattern of bland spindle cells with mild nuclear atypia, slightly eosinophilic cytoplasm, and artifactual paranuclear vacuoles, which are typical in stomach GISTs. Nuclear palisading, perivascular hyalinization, and fibrosis are occasionally observed [63]. Most GISTs are immunohistochemically positive for CD34, KIT (CD117), and DOG1 [63]. Nuclear β-catenin immunostaining is negative in the majority of GISTs [64]. DTFs are usually negative for CD34, but false positive immunostaining for KIT (CD117) has been documented in the literature [65]**,** while DOG1 is typically negative. Immunohistochemistry is very useful in distinguishing intraabdominal DTFs from GISTs.

The myxoid pattern of DTF was observed frequently in this study, appearing in up to 20 tumors (60%) with volumes ranging from 5 to 40%. This is in contrast with the findings of Zreik et al., who reported that the myxoid pattern was uncommon, occurring in only 27 cases (16%) with a comparable percentage of total tumor volume as our study [15]. We encounter a myxoid pattern in DTFR, raising a few differential diagnoses such as intra-muscular myxoma, low-grade myxofibrosarcoma, low-grade fibromyxoid sarcoma, and nodular fasciitis.

Intra-muscular myxoma is a benign, well-defined, non-infiltrative hypocellular tumor composed of bland spindle to stellate cells with no atypia or mitosis embedded in an abundant myxoid background. Myxoma is often immunohistochemically CD34-positive, rarely SMA-positive, and desmin- and S100-negative [1]. The literature contains limited information on the expression of nuclear β-catenin in intramuscular myxoma. β-catenin is expressed in 93% of infantile sinonasal myxomas as a separate entity [66]. More than 90% of intramuscular myxomas have a GNAS activating point mutation (WHO). One of the DTFs in this present cohort had an area of myxoid pattern that was similar to a myxoma, making it more difficult to make the proper diagnosis; thus, correlation with radiological, immunohistochemical, and molecular data is critical in difficult scenarios.

Myxofibrosaroma typically arises in the limbs of older people in subcutaneous areas. It has a wide range of morphology, ranging from low to high grade. Low-grade myxofibrosarcoma is characterized by multinodular, focally infiltrative hypocellular neoplasms composed of non-cohesive, spindled to stellate tumor cells with ill-defined, somewhat eosinophilic cytoplasms; enlarged, hyperchromatic nuclei; and uncommon mitosis. A distinguishing feature is the presence of conspicuous, elongated, curved, thin-walled blood arteries with perivascular tumors and/or inflammatory cell condensation. In the current study, focal mild-to-moderate nuclear atypia was observed in 67% and 15.15% of DTF, respectively, but no significant nuclear atypia or curvilinear blood vessels were found. Myxofibrosaroma does not have a specific immunohistochemical or molecular characteristic. Nuclear β-catenin immunostaining is infrequently observed in myxofibrosarcoma [67].

Low-grade fibromyxoid sarcoma (LGFMS) is one of the great mimickers of DTF. The tumor typically appears in the proximal extremities or trunk, but unusual places such as the abdomen and thoracic cavity, visceral organs, and cerebral sites have been documented [68]. Microscopically, LGFMS is distinguished by alternating collagenous hypocellular and cellular myxoid regions. Typically, there is an abrupt shift between these two areas. Tumor cells are bland, spindly, and occasionally plump, and they form short fascicles or whorls. Mitotic activity is usually low. Arcades of tiny and arteriole-sized arteries with perivascular sclerosis are visible. Occasional collagen rosettes with a central core of hyalinized collagen surrounded by a cuff of epithelioid-like tumor cells are found. These tumors always feature either FUS::CREB3L2 or FUS::CREB3L1 gene fusions and infrequently EWSR1::CREB3L1 fusions [69]. MUC4 immunohistochemical staining is an extremely sensitive and specific marker for LGFMS that is always negative for DTFs [70]. Furthermore, β-catenin is typically negative in LGFMSs [71]; hence, MUC4 and β-catenin immunohistochemical stains are useful tools for discriminating between those entities.

Zreik et al. found a nodular fasciitis (NF)-like pattern in DTF, which was observed in 12 tumors (36.3%) throughout the study [15]. Nodular fasciitis is a cellular lesion characterized by plump spindle-shaped cells with short fascicles and a storiform pattern, as well as focal discohesion, myxoid, and tissue culture-like appearance. Mitotic figures are common; however, there are no atypical forms. Extravasated erythrocytes, lymphocytes, and osteoclast-like giant cells are commonly found. SMA is typically positive in the spindle cells of NF in a tram-track pattern. SMA positivity can be observed in DTF; in this study, it was seen in 28 (85%) of the DTFs. Cases of DTFs with widespread nodular fasciitis-like patterns will be difficult to distinguish from NF. NF is distinguished by USP6 gene rearrangement [72] and is typically negative for β-catenin immunohistochemistry staining [47]; therefore, it will aid in identifying those entities in difficult cases.

At the molecular level, more than 85% of sporadic desmoid-type fibromatosis cases had missense mutations in exon 3 of the CTNNB1 proto-oncogene. These alterations mostly target the serine threonine phosphorylation sites at codons 41 and 45. Trautmann et al. found pathogenic non-CTNNB1 mutations in 18.2% of pediatric cases, including AKT1, ALK, IDH2, RET, and SDHD, compared with 8.8% in adult DTF cases, including AKT1, ALK, AR, EGFR, ERBB2, IDH2, KIT, KRAS, RET, and SDHA [31]. Meazza et al. investigated 16 sporadic cases of pediatric aggressive fibromatosis using NGS and discovered that, in addition to the CTNNB1 gene mutation, five cases (31%) had AKT1 E17K (25%), BRAF V600E (12%), TP53 R273H (6%), and RET V648I (6%) mutations [73]. They also discovered single-nucleotide polymorphisms (SNPs) in Q472H VEGFR2 (56%), I391M PIK3CA (31%), P72R TP53 (19%), F386L FGFR3 (12%), and M541L c-KIT (12%). The current study discovered three novel non-CTNNB1 gene mutations: BCL10, MPL, and RBM10. These mutations have been linked to a variety of malignancies, including hematopoietic, large intestine, lung, and urinary bladder cancers, among others. All of these findings point to DTF’s varied and complicated genetic makeup. They are most likely harmful in nature, although their significance is unknown.

In conclusion, this cohort study examined the histological and immunohistochemical aspects of DTF, uncovering the varied morphological characteristics of this tumor that could potentially lead to misdiagnosis. Hence, surgical pathologists ought to possess knowledge on this matter. Additionally, we provide previously unreported non-CTNNB1 gene mutations in sporadic cases of DTF, the significance of which remains unknown. Nevertheless, this study is limited by its retrospective nature and the small number of cases in this cohort. Hence, further research and examination are imperative to fully comprehend the clinical implications of these alterations and their viability for targeted therapeutic interventions.

## Figures and Tables

**Table 1:** Antibodies were used in the immunohistochemical analysis.

Antibodies	Clone	Source	Specific Antibody Concentration
β-catenin	β-catenin (14)	Ventana, REF 760-4242	2 µg/mL
CD34	CONFIRM Anti-CD34 (QBEnd/10)	Ventana, REF 790-2927	0.8 µg/mL
Desmin	CONFIRM Anti-desmin (DE-R-11)	Ventana, REF 760-2513	5 µg/mL
SMA	Actin, smooth muscle (1A4)	Ventana, 760-2833	0.03 µg/mL
S100	CONFIRM anti-S100 (4C4.9)	Ventana, REF 790-2914	10 µg/mL

**Table 2:** DTF anatomical site classification.

DTF Anatomical Classification *N* = 33	DTF Specific Site	Number of Specimen
Abdominal wall	Abdominal wall	6 specimens, 18.18%
Intra-abdominal		8 specimens, 24.24%
	Mesentery	5
	Pelvic mass	3
**Extra-abdominal**		19 specimens, 57.58%
	Left mandible	1
	Right lateral	1
	pharyngeal wall	1
	Right breast	1
	Left breast	1
	Left thigh	1
	Right shoulder	1
	Left side back	1
	Right neck	1
	Right shoulder mass subscapular	1
	Left shoulder	1
	Right groin	1
	Right parotid	1
	Central back of neck	1
	Back	1
	Submental	1
	Left axilla	1
	Right thigh	1
	Right leg	1

**Table 3:** Correlation of the histological pattern of desmoid-type fibromatosis (DTF) with recurrence.

The Mean of Histologic Pattern	Patients with Recurrence *N* = 7	Patients with no Recurrence *N* = 11	*p* Value
Conventional pattern	46%	63%	0.299
Hypercellular pattern	9%	12%	0.5413
Myxoid pattern	16.42%	10%	0.3673
Hyalinized/Hypocellular patten	24.28%	6.36%	0.0895
Staghorn/hemangiopericytomatous blood vessels pattern	4.28%	7.27%	0.3922
Nodular fasciitis-like pattern	2.85%	6.81%	0.5738
Keloid-like pattern	2.14%	2.72%	0.8247

## Data Availability

The data for this study will not be made publicly available due to privacy and ethical restrictions.
